# The classification of gastric antral vascular ectasia in cirrhotic patients by Versatile Intelligent Staining Technology

**DOI:** 10.1186/s43066-022-00198-9

**Published:** 2022-06-10

**Authors:** Randa Salah Eldin Abdelmoneim, Amr Aly Abdelmoety, Nahed Baddour, Perihan Salem, Marwa Metawea

**Affiliations:** 1 Internal Medicine Department, Faculty of Medicine, Alexandria University, Alexandria, Egypt; 2 Pathology Department, Faculty of Medicine, Alexandria University, Alexandria, Egypt

**Keywords:** Gastric antral vascular ectasia, White light endoscopy, Portal hypertensive gastropathy, Versatile Intelligent Staining Technology

## Abstract

**Background:**

Portal hypertensive gastropathy (PHG) and gastric antral vascular ectasia (GAVE) are two different pathologies that cause bleeding in cirrhotic patients. These two pathologies are still difficult to be distinguished by white light endoscopy (conventional), as they both appear as red spots in the gastric antral mucosa in the case of severe PHG. The aim of our study was to assess the efficacy of Versatile Intelligent Staining Technology (VIST) in comparison to histopathology in the diagnosis and classification of GAVE.

**Methods:**

A cross-sectional study included 50 patients with liver cirrhosis recruited from Alexandria Main University Hospital. Patients with connective tissue diseases and chronic kidney disease were excluded. All patients were examined by both conventional white light endoscopy (WLE) and image enhancement technology (VIST) using Sonoscape HD500 endoscope. GAVE was diagnosed as tortuous columns of ectatic vessels in the gastric antrum. Histopathological examination was used as the standard tool for the diagnosis of GAVE.

**Results:**

A total of 50 patients were included, 28 patients (56 %) were diagnosed as GAVE by pathology vs 22 (44 %) as non-GAVE. Twenty-three of 28 (78.6 %) cases of GAVE were detected by VIST. VIST had superior sensitivity than WLE in the detection of GAVE, 82.1 % vs 7.1 %, while WLE had higher specificity 95.5 % vs 59.1 % by VIST. There was statistical significance between VIST and pathology in the diagnosis of GAVE, *p*<0.035, but no statistical significance between WLE and pathology. VIST has identified two types of GAVE: focal in 12/28 cases and diffuse in 11/28, and five were not diagnosed by VIST.

**Conclusions:**

Versatile Intelligent Staining Technology could be used as an alternative tool to histopathological diagnosis of GAVE. GAVE can present as a focal group of ectatic vessels which adds a new class to GAVE classification that was previously misdiagnosed.

## Introduction

Gastric antral vascular ectasia (GAVE) is one of the causes of gastrointestinal bleeding. It represents a vascular dilatation in the muscularis mucosa of the stomach. It was found in association with liver cirrhosis, renal insufficiency, systemic sclerosis, and neoplasia [1, 2]. Recently, several studies have linked GAVE to metabolic syndrome [3]. Mostly, the patients present by iron deficiency anemia, or occult bleeding, and frank hematemesis [4].

The classification of GAVE consists of linear, punctuate, and recently nodular [5]. The punctuate or diffuse type is more common in cirrhotic patients, while the linear type is more common in systemic sclerosis [6].

Portal hypertensive gastropathy (PHG) is present in most of cirrhotic patients. It is characterized by vascular dilatation and mucosal edema. It is one of the major causes of bleeding in these patients.

PHG and GAVE are two different pathologies that cause bleeding in cirrhotic patients. These two pathologies are still difficult to be distinguished by white light endoscopy (conventional), as they both appear as red spots in the gastric antral mucosa in the case of severe PHG [7]. So, the definite diagnosis of GAVE depends on the histological criteria of the biopsy. By histopathological examination, GAVE may show vascular ectasia of mucosal capillaries, spindle cell proliferation (smooth muscle cell and myofibroblast hyperplasia), and peri-capillary fibrohyalinosis and most characteristically the presence of focal fibrin thrombi [8]. While in PHG, there is vascular dilatation of mucosal and submucosal capillaries without significant inflammation or fibrin thrombi [9].

The advances in endoscopic technology introduced narrow-band imaging (NBI) technology and its derivatives. NBI depends on the penetration properties of light by an optical filter placed in front of the light source. Other derivatives depend on digital image staining like Versatile Intelligent Staining Technology (VIST), which has the same concept of NBI, but instead of using an optical filter, digital processing of images to clarify the appearance of both vascular and mucosal patterns without dye application. It is easy to perform and allows for inspection of the whole endoscopic field with more accuracy by simply switching the button from the conventional view to the VIST view [10].

The objective of our study was to assess the role of the new technology VIST which depends on digital image staining to enhance the vasculature of the stomach and to estimate the prevalence of GAVE in cirrhotic patients.

## Patients and methods

The study was conducted in accordance with the provisions of the Declaration of Helsinki of the World Medical Association and was approved by the Institutional Review Board of the Alexandria Faculty of Medicine on 24 October 2019 with serial number 0201288. An informed consent was obtained from all patients included in the study.

### Patients

The patients were recruited from the outpatient clinic and Hepatology Unit ward in Alexandria Main University Hospital. All cirrhotic patients who were undergoing upper endoscopy, who met the inclusion criteria, were included in the study.

The inclusion criteria were:

- Aged 18 or above.
- confirmed diagnosis of cirrhosis and portal hypertension by ultrasonography.
- Patients with mucosal vascular lesions at the gastric antrum suspicious of GAVE of a linear striped form or a diffuse punctate form upon conventional endoscopy.

The exclusion criteria were:

- Connective tissue disorders like systemic sclerosis.
- Chronic kidney disease.
- Recent use of proton pump inhibitors in the last 14 days.
- History of gastric surgery or gastric cancer.
- Recent history of non-steroidal anti-inflammatory drugs intake.

### Sampling

Non-probability consecutive sampling in which every patient met the criteria of inclusion was selected till the required sample size was achieved.

### Endoscopy and biopsy sampling procedure

Then, an advanced inspection using VIST and targeted biopsy sampling of the red spots in the gastric antrum and body was performed at a time.

The type of endoscopy used was the HD-500 Video endoscope System by Sonoscape. It is classified as high-definition endoscopy with VIST. It has the advantage of allowing to perform endoscopy with a high-definition image, then in one click switched to VIST mode to assess the mucosal vascular architecture.

During the years 2019–2020, a total of 78 cirrhotic patients were assessed by conventional endoscopy, followed by VIST view. 50 patients who met the inclusion criteria were included. Further examination by VIST, characteristics of GAVE and PHG were recorded. GAVE appears as ring-type capillary dilatation while PHG features are a mosaic pattern with areas of hemorrhage around gastric pits [11, 12].

After that, the diagnosis was confirmed by biopsy. Biopsies were taken from the lesions and were examined by an expert pathologist unaware of endoscopy results. Further immunohistochemistry testing by CD61 a platelet marker was used to confirm the diagnosis [13]. The patients were divided into GAVE and non-GAVE groups.

## Results

The mean age of the patients was 57.60 ± 7.63 years. Sixty-four percent of them were males and 36% were females. Thirty-eight patients had hepatitis C-related cirrhosis (22 of them received direct antivirals agents and they achieved sustained virological response). Eight patients had nonalcoholic steatohepatitis and 4 patients had cryptogenic cirrhosis. Forty-four percent of the patients had diabetes. According to the Child-Pugh score, 26% were Child A, 56% were Child B, and 18% were Child C. By history, half of the patients had no history of bleeding, while the other half had attacks of bleeding (one case up to 8 attacks). On clinical examination, the most noticeable signs were splenomegaly and palmar erythema 62% and 60%, respectively. The laboratory data of these patients were documented (Table 1).

**Table 1:** Descriptive of the studied cases according to laboratory data (*n* = 50)

Laboratory data	Min.–Max.	Mean ± SD.	Median (IQR)
**Hemoglobin (g/dl)**	6.60–13.50	9.39 ± 1.72	9.10(8.0–10.5)
**Platelets (×10**^**3**^**/μl)**	37.0–363.0	114.48 ± 59.01	99.50(76.0–146.0)
**WBCs (×10**^**3**^**/μl)**	1.53–18.10	5.25 ± 3.14	4.15 (3.28–6.79)
**Total bilirubin (mg/dl)**	0.30–17.0	2.44 ± 3.34	1.60 (0.80–2.10)
**ALT(U/L)**	12.0–169.0	38.99 ± 30.57	32.50 (22.0–40.0)
**AST (U/L)**	13.0–163.0	54.33 ± 31.38	47.0 (34.0–65.0)
**Albumin (g/dl)**	1.40–4.0	2.94 ± 0.58	2.90 (2.60–3.30)
**Prothrombin activity (%)**	31.0–100.0	67.32 ± 15.87	67.70(54.30–78.0)
**INR**	1.0–2.49	1.36 ± 0.27	1.34 (1.16–1.50)
**Gastrin (pg/ml)**	29.50–846.0	162.77 ± 163.91	123.0(59.80–184.0)

### Endoscopic examination

#### WLE view

All patients underwent for conventional endoscopy and a meticulous examination of the antrum was done to search for erosions. The endoscopic findings by WLE were recorded (Table 2).

**Table 2:** Distribution of findings in the studied cases according to WLE (*n* = 50)

	No.	%
**Antrum**		
No erosions	4	8.0
Single lesion	15	30.0
Multiple	25	50.0
PHG	3	6.0
GAVE	3	6.0
**Body (PHG)**		
No	3	6.0
Mild	24	48.0
Severe	23	46.0
**Varices**		
No EV	11	22.0
Grade 1	3	6.0
Grade 2	8	16.0
Grade 3	28	56.0

#### VIST view

**Fig. 1:**
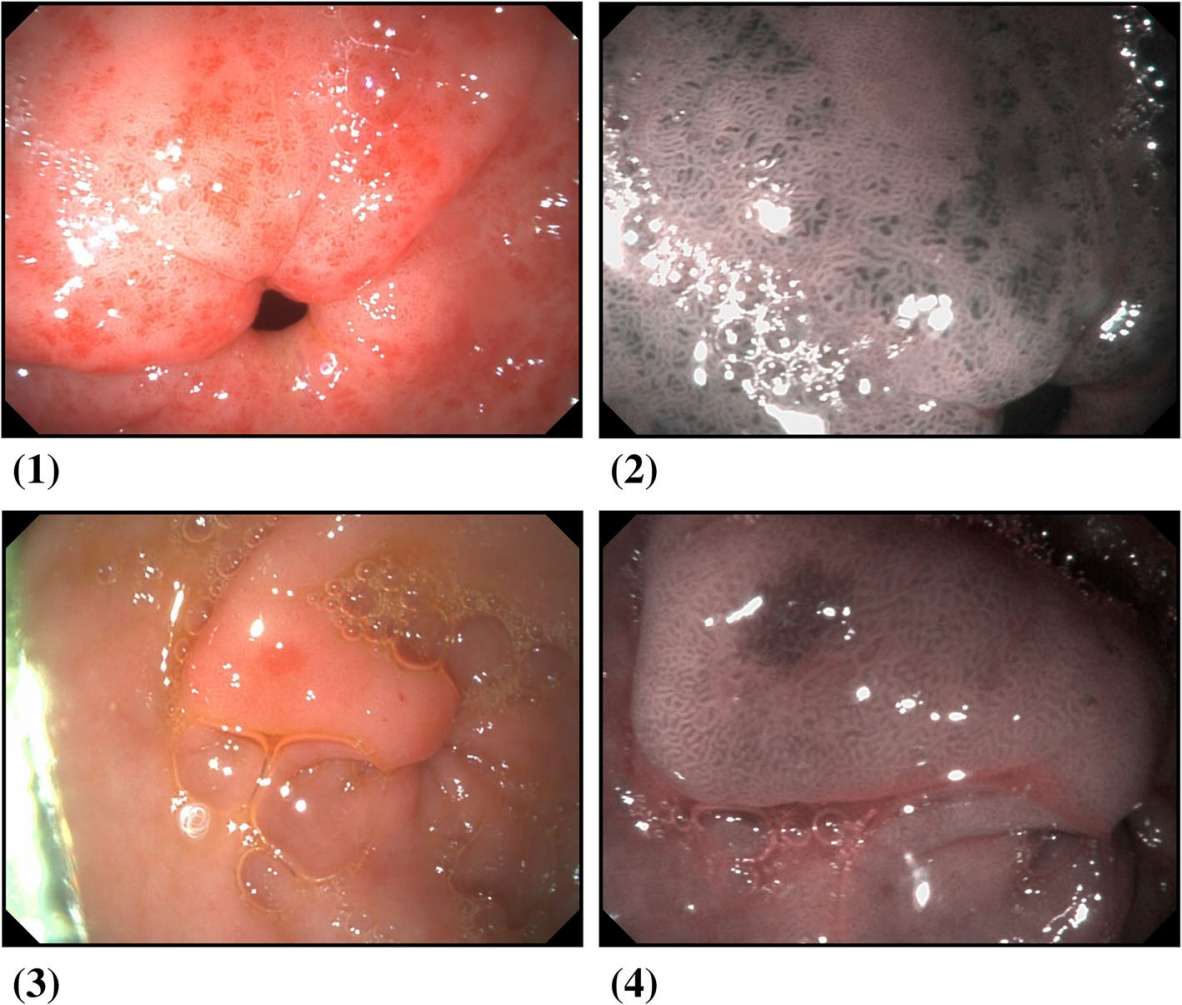
WLE and VIST view of two forms of GAVE found in the studied patients: (1&2) GAVE diffuse type and (3&4) GAVE focal type respectively

Endoscopic findings were that 70% (35 patients) were classified as having the endoscopic characteristics of GAVE (ring pattern), while the other 30% were classified as non-GAVE. In the body, PHG was detected in 94% of the patients (Fig. 1).

### Histopathological examination

**Fig. 2:**
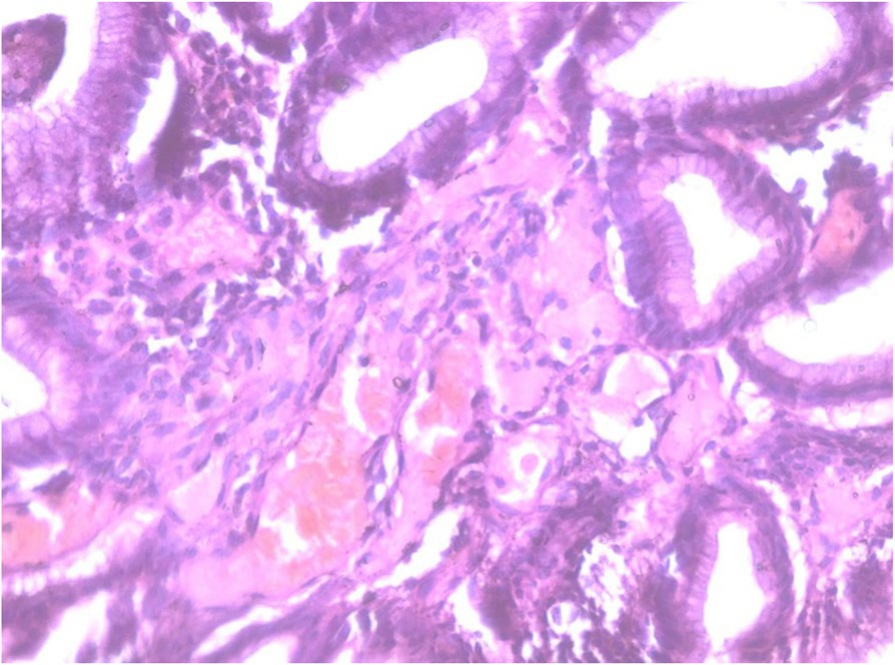
Gastric antral biopsy showing signs of GAVE; fibrosis of the lamina propria with fibrin thrombi in the small vessels (H & E, ×400)

Biopsies were taken from antral lesions and surrounding mucosa. They revealed different pathologies within the antrum. The highest prevalence was GAVE with 28 patients from the total of patients (56%), which VIST detected 23 of them (78.6%), followed by *H. pylori* with 19 patients, then 2 patients with dysplasia and only one patient with a diagnosis of PHG in the antrum. The diagnosis of types of GAVE visualized by VIST was confirmed. Among the group of GAVE patients, 13 patients were having one or two red spots that were seen as a focal area of GAVE and were proven by histopathology as GAVE (Fig. 2).

Then, immunohistochemistry using CD61 was applied on biopsies to detect fibrin thrombi in cases endoscopically suspected of GAVE but did not satisfy the histologic criteria necessary for its diagnosis. A single case showed fibrin thrombi that were not detected by H & E examination and therefore the diagnosis of GAVE was confirmed in this case.

The prevalence was estimated by the three different modalities. The highest prevalence was detected in 64% by VIST, followed by the pathological examination (56%) and the least prevalence was by WLE (6%).

VIST sensitivity was 82.1% in comparison to WLE 7.1% which illustrates the utility of VIST as a sensitive test for the detection of GAVE. While WLE remains higher in specificity with 95.5% in comparison to VIST (only 59.1%). The measure of agreement between pathology as a gold standard test and VIST had a *p* value of 0.035 (*p*≤0.05), which is statistically significant. According to the forms, VIST has succeeded to detect 12/13 of the focal GAVE cases (92.3%) and 11/15 of the diffuse GAVE cases (73.3%). VIST had sensitivity and specificity reached 92.3% and 92.8%, respectively for diagnosis of the focal GAVE with Kappa measure of agreement (0.85) (Table 3).

**Table 3:** Comparison and agreement of GAVE detection between WLE and VIST versus pathology as the gold standard

	Pathology results				Sp (%)	Sn (%)	FPR	FNR	McNemar ***p***	Kappa measure of agreement
	Negative biopsy (***N*** = 22)		Positive biopsy (***N*** = 28)							
	(−ve) Endoscopy	(+ve) Endoscopy	(−ve) Endoscopy	(+ve) Endoscopy						
**WLE**	**21**	**1**	**26**	**2**	**95.5**	**7.1**	**4.5**	**92.9**	<.001*	**K.023, p.701**
**VIST**	**13**	**9**	**5**	**23**	**59.1**	**82.1**	**40.9**	**17.9**	**.424**	K.421, p.035*

McNemar test for comparing proportion between VIST and pathology results

*WLE* white light endoscopy, *VIST* Versatile Intelligent Staining Technology, *Sp* specificity, *Sn* sensitivity, *FPR* false positive rate, *FNR* false negative rate, *K* Kappa Measure of agreement, *: statistically significant where *p* value≤ 0.05

## Discussion

The most noticeable finding in this study is the higher prevalence of GAVE (56%) than reported in previous studies (13%) [14, 15]. We reported that this percentage is probably due to the high prevalence of liver cirrhosis in Egypt. Other factors can contribute in the pathogenesis of GAVE and should be investigated like the role of metabolic syndrome [16]. Another finding was the presence of a type of GAVE that appeared as one or two red spots in the antrum. This form was detected by VIST view only and was confirmed by histopathology. It can be an early stage of development of diffuse GAVE or it may be a new subtype that was not covered in literature. According to Thomas et al., he has described only three phenotypes for GAVE: linear, diffuse, and nodular [5]. Thus, a new term could be introduced “focal GAVE”; this may be added to GAVE classification. This highlights the superiority of VIST over WLE in the detection of focal GAVE.

The efficacy of virtual chromoendoscopy has been proven in the diagnosis of GAVE over WLE without the need for invasive intervention in taking biopsy. VIST has succeeded in the diagnosis of 23/28 cases of GAVE. While WLE diagnosed only 2/28 of the cases. Our results were in consistence with Chang et al. who demonstrated the accuracy of NBI in the diagnosis of GAVE [12]. As PHG and GAVE are two different pathologies with different management, this could accelerate the diagnosis of GAVE and early management of bleeding.

The sensitivity of VIST was 82.1% in the diagnosis of GAVE versus WLE (7.1%). Which made VIST a good screening tool for the detection of GAVE among the patients with PHG. WLE has a specificity that reached 95.5%, but it only detected the cases of the typical endoscopic image, which were two cases only. While the specificity of VIST was lower (59.1%), VIST has succeeded to detect 12/13 of the focal GAVE cases (92.3%) and 11/15 of the diffuse GAVE cases (73.3%). The specificity decreased due to the presence of false positive cases in the diagnosis of diffuse form, mostly due to the presence of other etiologies as H Pylori, dysplasia, and one case of PHG. VIST had sensitivity and specificity that reached 92.3% and 92.8% respectively for diagnosis of the new subtype “Focal GAVE” with Kappa measure of agreement (0.85). Therefore, VIST can be a good test for the detection of focal GAVE. In the literature about the new endoscopic technology, GAVE was not widely covered. NBI, flexible spectral imaging color enhancement (FICE), and I-scan showed superiority over WLE in differentiating GAVE from PHG, but their number of patients was relatively smaller than in this study [12, 17, 18]. In addition, this study is considered the first to use VIST technology for the detection of GAVE.

The role of immunohistochemistry was complementary to histopathology. The diagnosis of GAVE depends on the presence of spindle cell proliferation, fibrohyalinosis, and most characteristically the presence of fibrin thrombi. The use of CD61 was important to identify subtle fibrin thrombi, not detected by H & E stain and thus to increase the detection of GAVE. It only reflects that GAVE can be present and not be able to be detected with regular histopathological examination. In the study, CD61 detected only one patient of the suspected GAVE by VIST in addition to the total diagnosed with H&E. Westerhoff et al detected more suspected cases of GAVE by using CD61, but he used conventional endoscopy to diagnose GAVE [13].

In conclusion, the efficacy of VIST a new technology derived from Narrow Band with a lower cost has been demonstrated in the diagnosis of GAVE in cirrhotic patients without the need for biopsy. Non-invasive vascular enhancement screening tool is mandatory, especially in cirrhotic patients with high bleeding tendency. VIST can be more reliable than WLE in the diagnosis of GAVE, and it could be used as a screening tool for the detection of GAVE, especially the focal form. Unfortunately, due to COVID 19 pandemic, the number of patients was limited. Larger or multicentric studies are needed in the future to assess its accuracy.

## Data Availability

The data is not publicly available. But it is available from the corresponding author upon reasonable request.

## Abbreviations

EV: Esophageal varices
FICE: Flexible spectral imaging color enhancement
GAVE: Gastric antral vascular ectasia
NBI: Narrow-band imaging
PHG: Portal hypertensive gastropathy
VIST: Versatile Intelligent Staining Technology
WLE: White light endoscopy
